# An Evaluation of the Genotoxicity and Subchronic Oral Toxicity of Synthetic Curcumin

**DOI:** 10.1155/2018/6872753

**Published:** 2018-07-16

**Authors:** Sreenivasa Rao Damarla, Rajesh Komma, Upendra Bhatnagar, Navin Rajesh, Sadik Mohmad Abdulhamid Mulla

**Affiliations:** ^1^Laurus Labs Pvt. Ltd., Plot No:DS1, IKP Knowledge Park, Genome Valley, Turkapally, Shameerpet, Hyderabad 500 078, India; ^2^Vimta Labs Limited, Pre-Clinical Division, Vimta Life Sciences Facility, Plot No. 5, MN Science and Technology Park, Genome Valley, Hyderabad 500 078, India

## Abstract

A battery of toxicological studies was conducted in accordance with international guidelines to investigate the genotoxicity and repeated-dose oral toxicity in rats of synthetic curcumin (VEAMIN 99, >99% purity). There was no evidence of mutagenicity in a bacterial reverse mutation test, whereas an in vitro mammalian chromosomal aberration test was positive for induction of chromosomal aberrations which is in line with results reported for natural curcumin. There was no evidence of genotoxicity in an in vivo mammalian micronucleus test. Synthetic curcumin did not cause mortality or toxic effects in a 90-day repeated-dose oral toxicity study at daily doses of 250, 500, or 1000 mg/kg body weight (bw)/day (administered by gavage in a split dose). The no observed adverse effect level (NOAEL) determined from the 90-day study was 1000 mg/kg bw/day for both male and female Wistar rats.

## 1. Introduction

Curcumin, a diarylheptanoid, is the most abundant of several natural curcuminoids found in the rhizomes of* Curcuma longa *(common name, turmeric) [1, 2]. Turmeric has been utilized for centuries in foods and as medicine in many parts of the world, including India and Malaysia [2–4]; recently, curcumin has been found to have biological activity as an anti-inflammatory, antioxidant, and anticancer agent [2–7].

Toxicological studies performed on various forms (e.g., extracts, modified extracts, and nanoparticles) and amounts of curcumin and other curcuminoids as mixtures have yielded varied results. Some studies in mice have shown signs of hepatotoxicity while others show no or equivocal toxicologically relevant effects [8]; similarly, studies in rats have largely shown an absence of toxicological concern, although some report equivocal evidence of carcinogenic activity [6, 9–12]. An in vitro study using human hepatoma G2 cells demonstrated that a low concentration of 2.5 *μ*g/mL of curcumin had no mutagenic effect; however, curcumin at higher concentrations (10–40 *μ*g/mL) appeared to induce mitochondrial and nuclear DNA damage in a dose-dependent manner [13]. In human clinical trials, curcumin at doses of 1125–8000 mg/day have been administered to participants with no toxic or adverse effects reported [2, 14].

We present herein a battery of Organization for Economic Cooperation and Development (OECD) compliant, in vitro, and in vivo toxicological studies on a novel bioidentical synthetic curcumin (VEAMIN 99) of >99% purity (Laurus Labs Ltd., India) as a contribution to the scientific knowledge of this individual compound.

## 2. Materials and Methods

### 2.1. Chemicals

All chemical reagents, solvents, pharmaceuticals, and other chemicals used in the studies were of analytical or pharmaceutical grade.

### 2.2. Test Article

The test article is a synthetic curcumin, a bright yellow to orange solid compound manufactured by Laurus Labs (Visakhapatnam, India). The sponsor provided curcumin of 99.4% purity from batch 25027-1VSP10410915, manufactured in August of 2015. Based on the results of preliminary solubility and compatibility tests with bacterial strains used in the bacterial reverse mutation test and human peripheral blood lymphocytes (HPBLs) used in the in vitro chromosomal aberration test, DMSO was chosen as the test article vehicle and vehicle control. Due to the physical characteristics of the test article, the vehicle and vehicle control for the in vivo mouse micronucleus test and 90-day repeated-dose oral toxicity test were 0.5% w/v carboxymethylcellulose sodium salt (CMC, Sigma Aldrich).

### 2.3. In Vitro Studies

#### 2.3.1. Bacterial Reverse Mutation Test

A bacterial reverse mutation test was conducted to investigate the mutagenic potential of synthetic curcumin according to the procedures described by Ames et al. [15], Green and Muriel [16], Mortelmans and Zeiger [17], Maron and Ames [18], and the test laboratory's standard operating procedures for preparations of frozen stock culture, raw data, and bacterial genotype confirmation. It was conducted in compliance with OECD 471 guidelines for the bacterial reverse mutation test [19] and Good Laboratory Practices (GLP) C(97)186/Final [20]. Bacterial tester strains* Salmonella typhimurium* TA98, TA100, TA102, TA1535, and TA1537 and S9 metabolic activation system (S9) were purchased from Molecular Toxicology, Inc. (NC, USA).

For the preliminary cytotoxicity assay, test solutions were prepared by dissolving curcumin in DMSO to achieve concentrations of 16.0, 5.0, 1.6, 0.5, 0.16, 0.05, and 0.016 mg/mL. For the mutagenicity assay, test solutions were prepared by dissolving the test item in DMSO to achieve concentrations of 5.0, 1.6, 0.5, 0.16, and 0.05 mg/mL. All test item preparations and dilutions were carried out under sterile conditions. S9 mix (cofactors and liver homogenate, 5% v/v) and positive controls were prepared freshly on the day of the experiment. Sodium azide and mitomycin C were diluted in water; all other positive controls were diluted in DMSO.

A preliminary cytotoxicity assay was performed utilizing the plate incorporation method in triplicate by exposing tester strains TA98 and TA100 with and without metabolic activation to the following concentrations of the test article: 1.6, 5.0, 16.0, 50.0, 160.0, 500.0, 1600.0, and 5000.0 *μ*g/plate. Positive controls for the experiments without S9 were 2-nitroflourine (25.0 *μ*g/plate) for TA98, sodium azide (20.0 *μ*g/plate) for TA100 and TA1535, and 9-aminoacridine (50.0 *μ*g/plate) for TA 1537 and mitomycin C/ametycine (0.25 *μ*g/plate) for TA102. The positive control for all of the experiments with S9 was 2-aminoanthracine (20.0 *μ*g/plate).

The mutagenic assay was performed utilizing the plate incorporation method, in triplicate, by exposing tester strains TA98, TA100, TA102, TA1535, and TA1537, with and without S9, to the following test article concentrations: 5.0, 16.0, 50.0, 160.0, 500, and 1600.0 *μ*g/plate. All treated plates were incubated at 37 ± 2°C for 68:25 (hours:minutes) in the preliminary cytotoxicity test and 66:10 (hours:minutes) in the mutagenicity assay after which the plates were manually examined for background lawn inhibition, precipitation, and revertant colonies.

A result was considered positive if

- there was at least a 2-fold increase (for TA100, TA102, and TA98) or 3-fold increase (for TA1535 and TA1537) in the mean revertants per plate of at least one of the tester strains over the mean revertants per plate of the appropriate vehicle control;
- the increase in the mean number of revertants per plate was accompanied by a dose response in a minimum of 2–3 concentrations.

#### 2.3.2. In Vitro Mammalian Chromosomal Aberration Test

The in vitro mammalian chromosomal aberration test was conducted to evaluate the ability of curcumin and/or its metabolites to induce structural chromosome aberrations in cultured HPBL. It was performed in compliance with OECD 473 [21] and GLP C(97)186/Final [20].

Test article formulations were prepared on the day of treatment by diluting stock solution with DMSO to achieve the test concentrations. HPBLs were obtained by drawing blood from healthy, young, nonsmoking males with no known illness or recent exposure to genotoxic agents and subsequently pooling and culturing blood in Roswell Park Memorial Institute Medium, with 15% Fetal Bovine Serum (FBS). Whole blood cultures were incubated at 37 ± 2°C in a humidified environment.

Positive controls were mitomycin C/ametycine, dissolved in water to a concentration of 0.25 *μ*g/mL for experiments without metabolic activation, and cyclophosphamide, dissolved in water to a concentration of 12.5 *μ*g/mL for experiments with metabolic activation.

A preliminary cytotoxicity assay was performed to determine the test concentrations for the chromosome aberration assay. HPBL cultures were exposed to the test article with and without metabolic activation at concentrations of 1.9, 3.9, 7.8, 15.6, 31.3, 62.5, 125.0, and 250.0 *μ*g/mL for four hours; additional HPBL cultures were continuously exposed to the same concentrations without metabolic activation for 22 hours. Experiments for all test groups including the vehicle control were performed in duplicate. At least one thousand cells in each culture were analyzed for mitotic index (MI; number of mitotic cells/total number of cells scored, expressed as a percentage). Cytotoxicity was defined as a reduction in MI to 45 ± 5% of the vehicle control.

The chromosome aberration assay consisted of two independent, concurrent experiments, a short-term exposure assay and a continuous exposure assay. In the short-term exposure assay, cells were exposed to the test article at concentrations of 10.0, 20.0, and 40.0 *μ*g/mL in the absence of metabolic activation, and to concentrations of 6.3, 12.5, and 25.0 *μ*g/mL in the presence of S9 metabolic activation and to corresponding positive and negative controls, and incubated. Following incubation, all cultures were washed with plain media and placed into fresh culture medium with 15% FBS to continue incubation until harvest.

In the continuous exposure experiment, cells were exposed to the test article at concentrations of 6.3, 12.5, and 25.0 *μ*g/mL, vehicle, and positive controls, and incubated in the absence of metabolic activation. Culture media was changed at the time of cell harvest. The pH was measured before and after all experiments

Approximately 20 hours after exposure initiation in all experiments, 0.1 mL of colchicine was added to arrest mitosis. Approximately 2.5 hours after application of colchicine (approximately 1.5 normal cell cycle lengths from initiation of treatment) cells were harvested and chromosome slides were prepared for analysis.

Slides were coded and scored blind and at least 1000 cells from each group were evaluated for MI. Scoring occurred on the basis of good chromosome morphology and only cells with equal numbers of centromeres and modal numbers (46 ± 2) were analyzed. Three hundred metaphases (150 from each duplicate) were evaluated for structural chromosome aberrations. The percent of polyploidy and endoreduplication was calculated by evaluating 250 metaphases per culture. Gaps were recorded separately but were not included in the total aberration frequency as gaps are considered achromatic lesions similar to nucleolar constrictions, which are easily broken by the pressure exerted during slide preparation [22] or most often the result of a single stranded DNA break, which is a reversible phenomenon as DNA has the innate capability to repair such aberrations [23].

The test was considered positive if a significant increase in the number of cells with chromosome aberrations was observed at one or more test concentrations and the increase was dose-dependent. The test was considered negative if none of the above criteria were met under all experimental conditions.

### 2.4. Animal Studies

The Institutional Animal Ethics Committee (IAEC) of Vimta Labs approved the in vivo mouse micronucleus test and 90-day repeated oral dose study protocols. The ethical practices set forth by the Committee for the Purpose of Control and Supervision on Experiments on Animals (CPCSEA) were followed throughout studies.

#### 2.4.1. In Vivo Mouse Micronucleus Test

The in vivo mouse micronucleus test was conducted to evaluate the genotoxic potential of synthetic curcumin to induce the formation of micronuclei in polychromatic erythrocytes (PCEs) in the bone marrow of mice. The study was conducted in compliance with OECD 474 (2014) [24] and GLP C(97)186/Final [20].

On the day of treatment, the test article was suspended in 0.5% w/v carboxymethylcellulose (CMC) sodium salt in water (Milli-Q, for injection) under sterile conditions to achieve concentrations of 25, 50, and 100 mg/mL and was administered at a dose volume of 10 mL/kg body weight (bw). Cyclophosphamide served as the positive control and was prepared on the day of treatment by dissolving 30.09 mg of cyclophosphamide monohydrate in deionized water to a concentration of 3 mg/mL. 0.5% w/v CMC sodium salt solution served as the vehicle control.

Seven-to-nine-week-old male and female Swiss Albino mice weighing 22.27–33.22 grams were utilized for this study. The mice were acclimatized, observed, and examined for a period of 5 days, per Vimta Labs' standard operating procedures, to confirm that the animals were in good health. Animals were housed in groups of up to three in sterilized suspended polycarbonate cages with laboratory bedding material; the room temperature was 21.1–22.9°C with 41.0–62.0% relative humidity and 12-hour light-dark cycles. All animals had access to standard pellets (Harlan Laboratories) and water* ad libitum*.

Animals were randomized into groups of 5 animals/sex/group at dose levels of 500, 1000, and 2000 mg/kg bw/day and negative and positive control groups. The test article and vehicle control were administered for two consecutive days, 24 hours apart, in divided doses (about 1–2 hours apart) by oral gavage with the dose volume maintained at 20 mL/kg bw. The positive control was administered 24 hours prior to sacrifice in a single dose of 30 mg/kg bw by gavage with dose volume of 10 mL/kg bw. Animals were observed for clinical signs at the time of dosing, one hour after treatment, and once daily thereafter, while being observed twice daily for mortality or moribund condition. Body weights were recorded on the day of receipt, before randomization, on treatment days, and the day of sacrifice. Bone marrow samples were collected from both exposed femurs of each animal 24 hours after the last dose. Three slides per animal were prepared, blind coded, and examined for incidence of micronucleated cells. A minimum of 4000 PCEs were scored per animal and the frequency of micronucleated PCEs (MNPCEs) was reported as percent of MNPCEs. The proportion of PCE of total erythrocytes (TE) was determined for each animal by counting a total of at least 500 erythrocytes.

Criteria for a positive result were as follows:

- At least one of the treatment groups exhibits a statistically significant increase in the frequency of micronucleated immature erythrocytes compared with the concurrent negative control.
- This increase is dose-related at least at one sampling time when evaluated with an appropriate trend test.

#### 2.4.2. 90-Day Repeated-Dose Oral Toxicity Studies in Rats

The 90-day study was conducted to evaluate the potential toxicity profile and target organs of repeated exposure to synthetic curcumin in male and female Wistar rats and to determine the no observed adverse effect level (NOAEL). The study was conducted in compliance with OECD 408 (1998) [25] and OECD GLP, C(97)186/Final [20].

The test article was suspended in 0.5% w/v CMC sodium salt solution in water (Milli-Q, for injection) to achieve concentrations of 12.5, 25, and 50 mg/mL. The reference item, a natural curcumin powder containing 97.9% w/w total Curcuminoids of Curcumin (80.1%), demethoxycurcumin (15.4%), and bisdemethoxycurcumin (2.4%) (Lot #15004819, Plant Lipids Ltd., India), was included for comparison of toxicological profiles between the curcumin test article and a natural curcumin with a different purity level under the same test conditions and was prepared in the same manner as the test article. All test article formulations were analyzed by high-performance liquid chromatography in the first, seventh, and last weeks of the treatment period and all results fell within the qualifying limit of 85–115% of curcumin. Formulation stability was tested and found to be stable for 48 hours at room temperature.

Following a detailed clinical exam on the fifth day of acclimatization, Wistar rats (Vivo Bio Tech Limited, Hyderabad, India), 5–7 weeks old, and weighing 170.83 ± 12.95 g (males) and 137.14 ± 10.37 g (females) were randomly divided by zigzag manual method based on body weight, into 5 groups of 20 rats/sex/group (three test article dose groups, one reference item group, and one control group). Animals were housed in same-sex pairs in sterilized polycarbonate cages in which they had access to reverse osmosis filtered water and certified standard pelleted laboratory animal diet (Envigo, Harlan Laboratory, USA)* ad libitum* except during fasting days when they received water only. Throughout acclimatization and treatment periods, animal rooms were maintained at 20.8–23.7°C and 38–68% relative humidity with light/dark cycles of 12 hours.

Doses and the divided-dose (bid) administration regimen were chosen based on solubility trials (single-dose suspensions were too viscous to administer) and a 14-day repeated-dose oral toxicity, non-OECD/GLP compliant dose range-finding study on Wistar rats (doses were 500, 1000, and 2000 mg/kg bw/day, administered bid). There were no toxic changes in the assessed parameters of the 14-day study and no test article related changes other than stool color. Therefore, it was concluded that the NOAEL for the test article after 14-day repeated-dose administration was 2000 mg/kg bw/day (1000 mg/kg bw bid), the highest dose tested. Although there were dose-dependent increases in absolute and relative liver weights in males of the 1000 and 2000 mg/kg bw/day groups, these findings were not considered toxicologically relevant due to lack of correlating clinical chemistry, gross pathological, or histopathological findings. Due to concern of greater organ weight increases with longer-term treatment, the following doses were chosen for the 90-day repeated-dose oral study: 250 mg/kg bw/day (125 mg/kg bw bid), 500 mg/kg bw/day (250 mg/kg bw bid), and 1000 mg/kg bw/day (500 mg/kg bw bid). The reference group received 1000 mg/kg bw/d (500 mg/kg bw bid) and the vehicle control received an equivalent volume of vehicle formulation, 0.5% CMC in sterile water. Each dose was administered by gavage, at least five hours apart, at a volume of 10 mL/kg bw, based on the most recent recorded weight of the animals.

Animals were observed for morbidity and mortality twice daily. General clinical observations occurred daily, and detailed clinical examinations occurred before randomization and weekly thereafter throughout the treatment period. Body weights for all groups were measured prior to gavage on day 1 and weekly until and on the day of sacrifice. Functional observations of all animals in the study took place during week 13 and consisted of observations of the animals in their home cage (e.g., body position), during handling (appearance of skin, fur, hair coat, piloerection, palpable mass, lacrimation, eye prominence, salivation, nasal discharge, and feces color and consistency and respiration), in the open field (gait, stereotypies, convulsions, tremors, pinnae response, palpebral closure, pupil reflex, approach response, touch response, and auditory, visual, and proprioceptive stimuli), and for neuromotor activity (grip strength, rearing, arousal, and nociceptive test).

Cage-wise food consumption was recorded weekly and calculated as the difference between food offered (grams) and food left over (grams) divided by the number of rats in the cage. Ophthalmologic examination was performed on all animals prior to the treatment period and on high-dose and control groups during the last week of the study.

After termination of treatment, three fasting blood samples from each animal were collected from the retroorbital plexus under isoflurane anesthesia for measurement of hematology, clinical chemistry, and coagulation parameters. Urine was collected at the end of the treatment period on day 91 for macroscopic and microscopic examination. On day 91, all animals were weighed and sacrificed (by CO_2_ asphyxiation) and underwent gross pathological examination after which absolute and relative organ weights were determined. Histopathological examination was performed on all preserved tissues of high-dose test and reference groups and vehicle control animals. Lung tissue of all low-dose and middose animals was examined following observations of histopathological findings in lungs of the high-dose group.

### 2.5. Statistical Analyses

Statistical analyses were performed using SAS® 9.2, Enterprise Guide version 4.3 for Windows (SAS Institute Inc., Cary, NC, USA). Per the test guidelines, the bacterial reverse mutation test results were interpreted based on the criterion of statistically significant changes; thus, no further statistical analysis was conducted. For the in vitro mammalian chromosomal aberration test, the Cochran-Armitage test for linear trend and Fisher's Exact Test were used to compare the percentage of cells with aberrations in treated cells to the results for the vehicle control. The number of aberrations in the treatment and positive control groups were compared to the corresponding negative control, and all groups were compared to laboratory historical data. For the mouse micronucleus test, the Cochran-Armitage test was used for linear trend and Fisher's Exact Test was used to compare the frequency of MNPCEs among the TEs in the treatment groups to the vehicle controls. Kruskal-Wallis Nonparametric Analysis of Variance (ANOVA) test was used to compare the ratio of PCEs to TE (PCE/TE) of the vehicle control group to the treatment groups. In the 90-day repeated-dose oral toxicity study, the D'Agastino and Pearson Omnibus tests were used to confirm the normality of the data. Normal data were tested with Levene's test for homogeneity of variance. Nonhomogenous data were appropriately transformed before analysis. Student's* t*-test was used to compare the high-dose test article and reference groups. Additional statistical testing for hematology, clinical chemistry, and coagulation results were analyzed by K-S test for normality, Bartlett's test for homogeneity, and ANOVA followed by Dunnett's multiple comparison tests using GraphPad Prism. The Kruskal-Wallis test followed by Mann–Whitney test was used to analyze nonhomogenous data. All statistical tests were performed at 5% and 1% levels of significance.

## 3. Results

### 3.1. Bacterial Reverse Mutation Test

Based on precipitation observed in the preliminary cytotoxicity assay, the highest test concentration used in the mutagenic assay was 1600.00 *μ*g/plate. Evaluation for mutagenicity revealed no 2-fold or greater increases for TA100, TA102, or TA98 and no 3-fold or greater increases for TA1535 or TA1537 in mean revertants per plate. Also, there were no dose-related increases in the mean number of revertant colonies in any of the tester strains at any test concentration, 5.0–1600.00 *μ*g/plate, with or without S9 compared to vehicle control (see Tables 1 and 2).

### 3.2. In Vitro Mammalian Chromosomal Aberration Test

Based on the cytotoxicity criteria, the short-term exposure concentrations were 6.3, 12.5, and 25.0 *μ*g/mL in the presence of S9 and 10.0, 20.0, and 40.0 *μ*g/mL in the absence of S9. The resulting percent reduction in MI in the presence of S9 was 16, 36, and 47%, respectively, and in the absence of S9 was 4, 25, and 41%, respectively, compared to vehicle control. The only group with a statistically significant increase in chromosome aberrations was the 25.0 *μ*g/mL group with S9, both including and excluding gaps; both groups also showed dose dependence (see Tables 3 and 4).

In the continuous exposure experiment, the test concentrations were 6.3, 12.5, and 25.0 *μ*g/mL and resulted in MIs of 25, 33, and 51%, respectively, compared to vehicle control. Counts of chromosomal aberrations including and excluding gaps showed no statistically significant increases in any test group compared to vehicle control. The positive control showed the expected increase in frequency of aberrant cells compared to control. The pH of the treatment medium at all test concentrations in both the short- and long-term exposure experiments was comparable to that of the vehicle control throughout the respective experiments.

### 3.3. In Vivo Mouse Micronucleus Test

Test article administration resulted in no mortality or treatment related changes in clinical signs or bodyweight in any of the test group or control animals. All animals appeared normal after dosing and remained healthy until the time of sacrifice.

After two administrations of the test article 24 hours apart at concentrations of 500, 1000, and 2000 mg/kg bw bid, there were no significant reductions compared to control in the ratio of PCEs to TE observed at any of the test article concentrations. Similarly, there were no statistically significant increases in the frequency of MNPCEs in any of the test article groups compared to vehicle control. Positive control treatment resulted in the expected increases in the incidence of MNPCEs, inducing a statistically significant increase compared to controls. The vehicle control group count for MNPCEs remained within historical control laboratory values (see Table 5).

### 3.4. 90-Day Repeated-Dose Oral Toxicity Study in Rats

There were no mortalities in any of the treatment, control, or reference groups at any time during the study period. Daily cage side observations, weekly clinical examinations, and the functional observation battery revealed no differences between test article, reference, and control groups other than yellow color changes of the feces, tails, and fur (see Table 6). In males and females of the high-dose and reference groups, fecal color changes were observed from Days 13 and 15 and tail color changes from Days 21 and 19, respectively, through the end of the treatment period. Fur color changes were observed in the high-dose and reference group males starting on Day 52 and reference group females from Day 51, through the end of the study. Fecal color changes were observed in middose males and females from Days 49 and 47, respectively, through the end of the study. No abnormalities were detected upon ophthalmological examination of the vehicle control, high-dose, or reference item groups; thus, animals in the low-dose and middose groups did not undergo this examination.

Mean body weights were similar in the test article and reference item groups compared to controls throughout the study. Additionally, mean body weights of the test article groups and reference group did not differ significantly from one another (see Table 7). Sporadic statistically significant increases in feed consumption compared to vehicle control were observed in males of the middose (Days 1–2 and 29–30) and high-dose (Days 7–8) test groups and in low- (Days 43–44) and high-dose (Days 43–44 and 85–86) test group females. Sporadic statistically significant decreases in feed consumption were observed in low-dose test article (Days 50–51) and reference group males (Days 1–2, 71–72 and 78–79) and in low-dose (Days 36–37) and middose (Days 1–2, 36–37, 57–58, and 64–65) females. Feed consumption in high-dose males was sporadically increased with statistical significance over the reference group males while in high-dose females feed consumption was similar to the reference group over the course of the treatment period (see Table 8).

There were some statistically significant differences compared to vehicle control with and without dose-dependency in hematological measures in the test and reference group males while there were sporadic statistically significant results for low-dose and middose group females (see Table 9). High-dose group males showed statistically significant increases in white blood cells (WBC) and lymphocytes compared to the reference group while there were no significant differences among high-dose and reference group females in any hematology measures. Sporadic statistically significant differences compared to control were observed in several clinical chemistry measures in the male test groups and in female test and reference groups (see Table 10). Statistically significant increases in the high-dose group males compared to reference group males were observed for chloride and phosphorus and in high-dose females compared to reference group females for sodium and chloride.

Several statistically significant changes compared to control in coagulation parameters were observed in male and female test article and reference groups (see Table 11). There were no statistically significant changes in macro- or microscopic urinalysis results (see Table 12).

Compared to control in any of the male or female test and reference groups (data not included), no treatment related changes were seen in urinalysis in test and reference item treated animals.

Sporadic statistically significant increase in absolute organ weights of the heart, brain, kidneys, testes, and epididymides were observed in the middose group males. Spleen weights were significantly decreased in reference group males compared to vehicle (see Table 13). A statistically significant decrease in liver weight in middose females and significant increases in adrenal weights in high-dose and reference group females were observed. Statistically significant increases in relative organ weights (body:organ) were observed in kidneys of low-dose, middose, and reference group males and in the liver of high-dose and reference group males (see Table 14). For females in the low-dose group, statistically significant increases were observed in relative organ weights of the lungs and spleen. Several sporadic gross pathological findings were observed in individual males in the vehicle control group and in test article groups, whereas findings in the reference group were isolated to the lungs (see Table 15). Few gross pathological findings were observed among female animals.

Histopathological examination in males and females revealed several lesions present in individual animals in the control, high-dose, or reference group, while several findings were present at the same frequency or were more frequent in the vehicle control group compared to the high-dose or reference group (see Table 16).

## 4. Discussion

In the performed bacterial reverse mutation test, both with and without S9, the test item was considered nonmutagenic. In the in vitro chromosomal aberration test, the test article did not induce structural chromosome aberrations in cultured HPBL in any of the tested concentrations in the short-term and continuous exposure experiments without metabolic activation. However, there was a dose-dependent increase with S9 in the short-term experiment, by 0.34, 1.67, and 4.33% in the frequency of aberrant cells in the 6.3, 12.5, and 25.0 *μ*g/mL treated cells, respectively, compared to vehicle control. The increased frequency in the 25.0 *μ*g/mL treated cells was statistically significant. Based on the positive results criteria for this test, it was concluded that the test article was clastogenic in cultured human peripheral blood lymphocytes under the conditions of this test. The test article did not induce micronuclei in the bone marrow of mice and was considered to be nonmutagenic under the conditions of the performed micronucleus test.

During the 90-day study, there were no mortalities in any of the five groups. The yellow discoloration in the feces and on the fur and tail of the middose, high-dose, and reference animals was attributed to the color and volume of the test article with the yellow color on the tail and fur resulting from external contact with the discolored feces and urine. The discoloration was not considered toxicologically relevant. There were no statistically significant changes in body weights throughout the study and no abnormalities were observed on ophthalmological examination. There were statistically significant changes in feed consumption; however, the changes were sporadic and minimal and did not affect the body weight of the animals. Therefore, the differences observed were not considered toxicologically relevant. There were no abnormalities observed in the functional observations of the animals in their home cages, during handling, in the open field, or with neuromotor activities.

Statistically significant changes were observed in hematology parameters among the test groups and reference group males. Significant decreases in hemoglobin and mean corpuscular hemoglobin concentration, increases in red cell distribution width and mean corpuscular volume, changes in mean platelet volume, and decreases in WBCs, monocytes, lymphocytes, eosinophils, and neutrophils showed no dose-dependency and remained within or marginal to historical control ranges; thus, these changes were not considered test article related and were attributed to normal variation. Statistically significant dose-dependent decreases in RBC and monocytes, compared to control, were observed in the male test groups as well as the reference group and the high-dose group mean corpuscular hemoglobin was statistically significantly increased compared to control with apparent dose relation; however, as all of the measures remained within biological range, the changes were considered incidental and within normal variation. Other than statistically significant increases in high-dose males compared to the reference group for WBC and lymphocytes, hematology measures for high-dose and reference group males were similar. Several slight but statistically significant increases and decreases in hematology parameters were observed in the low-dose and middose female groups; as the changes were low in magnitude and there were no significant changes in the high-dose group females (no dose relationship), the changes in the low-dose and middose females were considered incidental and of no toxicological consequence. A statistically significant decrease in reticulocytes was observed in the high-dose females compared to the reference females and was also considered an incidental change with measures remaining within the historical range.

Statistically significant differences in clinical chemistry measures in test group males were without dose relation, were present only in the low-dose and/or middose groups, and/or remained within or marginal to historical control ranges; therefore, the changes were considered within the normal variation of the animals and of no toxicological significance. Phosphorus and chloride were statistically significantly increased compared to the reference group with phosphorus which also significantly increased compared to vehicle control with potential dose relation. However, all measures for phosphorus and chloride remained within biological ranges and differences were present without correlating changes in gross pathology or histology.

Similarly, clinical chemistry results for female groups revealed statistically significant differences among several measures. However, the changes were not dose-related, remained within or marginal to historical control values, and/or were considered nonadverse (e.g., lowered cholesterol); thus, they were considered within the normal biological range of the animals.

Coagulation parameter results revealed statistically significant decreases in prothrombin time (PT) for middose and high-dose males that were marginal to the historical range. Since the decrease in fibrinogen occurred simultaneously with a faster clotting time (PT) and no significant change was seen in platelets, the changes are not considered of toxicological significance. Fibrinogen levels in low-dose males were statistically significantly decreased compared to control with PT, activated partial thromboplastin time (APTT), and platelets remaining unaffected; thus, the change is not considered biologically relevant. The high-dose group result for PT was significantly decreased compared to the reference item and was considered incidental. Additionally, all coagulation results for males fell within or marginal to the historical ranges and were considered within the range of normal variation.

Coagulation parameters in females revealed no significant changes in fibrinogen levels and only a statistically significant decrease for the middose group for APTT. PT was statistically significantly increased in the low-dose, middose, and reference groups without dose relationship. The absence of dose relationship, along with no change in platelet counts, the nonadverse change in APTT, and all results falling within historical controls, leads to the conclusion that the changes are not toxicologically relevant.

Urinalysis results contained no significant findings. The changes in several absolute organ weights in male groups (heart, brain, kidneys, spleen, testes, and epididymides) showed dose-unrelated, statistically significant increases with no associated changes in gross pathology or histopathology; thus, they were considered incidental findings. In females, liver weight was statistically significantly decreased in the middose group and the adrenal glands were statistically significantly increased in the high-dose group. The changes lacked correlating gross pathological or histopathological findings; thus, the changes were considered incidental.

Relative organ weight changes for male kidney weights were not dose-dependent and cooccurred with only one gross kidney lesion each in the low-dose and middose groups. Increases in liver weight relative to body weight in males have apparent dose relationship with nonsignificant increases also seen in the low-dose and middose groups; however, the increases occur in the absence of related gross pathological or clinical chemistry findings and the only hepatic histopathological finding (cystic, focal degeneration) was in a single high-dose male. Thus, the increases in relative kidney and liver weights are not considered toxicologically relevant. Since the only statistically significant changes in organ weight ratios for females were increases in the lungs and spleen of the low-dose group, the findings were considered incidental.

The yellow discoloration of the lungs in test article group males and females and in reference groups, along with the histopathological findings (infiltration of foamy macrophages, chronic inflammatory foci, chronic inflammation and interstitial fibrosis, foreign body granuloma, and osseous metaplasia), was consistent with findings associated with oral gavage error, spontaneous lesions, and/or aspiration of the test and reference solutions [26, 27]. In the absence of correlating histopathological or urinalysis findings and due to the small number of animals affected, the macroscopic kidney changes (cyst and nephrosis) were considered incidental [27, 28]. Histopathological findings in the testes (atrophy, degeneration of seminiferous tubules, and interstitial bilateral edema) and epididymides (epithelial vacuolation, sperm granuloma, or oligospermia) occurred with greater or equal frequency in the vehicle control group compared to the high-dose or reference groups; thus, the male reproductive organ findings were considered individual, incidental occurrences in experimental rats [28]. The remaining histopathological findings in males (in the liver, kidneys, heart, stomach, mesenteric lymph nodes, spleen, thymus, prostate, and skeletal muscle) and in females (liver, kidneys, heart, mandibular lymph node, pancreas, adrenal glands, spleen ovaries, uterus, urinary bladder, and pituitary gland) occurred in individual animals only (some findings were present in the vehicle control and/or reference group only) and were considered sporadic, incidental, and unrelated to the test article [26, 28, 29].

## 5. Conclusions

In the studies reported herein, the test article, synthetic curcumin, was found to be nonmutagenic in the bacterial reverse mutation test, positive for clastogenic activity in the in vitro chromosomal aberration test, and nongenotoxic in the mammalian micronucleus test, with a NOAEL of 1000 mg/kg bw/day in this 90-day repeated-dose oral toxicity study. Natural curcumin has been shown to induce chromosomal aberrations in cells at various stages of cell division at levels of 5–10 *μ*g/mL or more [30–36]. Investigations into why this positive result occurs in vitro alongside other in vitro and in vivo animal studies that result in no genotoxicity suggest that one mechanism for chromosomal aberrations is the potential of curcumin to generate and/or promote hydroxyl radical formation under the experimental conditions of the chromosomal aberration test. Other studies have investigated the radical scavenging/promoting activity of curcumin, a polyphenol, under test conditions such as the Fenton reaction, finding that curcumin (and other phenolics) at low doses can promote hydroxyl radical formation but at high doses can protect against hydroxyl radical formation [30, 37, 38]. Araújo et al. suggest that curcumin may act by inhibiting chromosomal damage repair, thus exacerbating chromosomal damage [32, 33]. In the context of this current battery of toxicology tests on curcumin, a positive result in one in vitro test alone does not necessarily lead to the conclusion that a substance is genotoxic in general, especially when in vivo tests (in this case, the mouse micronucleus test) show nonmutagenic results [31]. In conclusion, the negative results of the bacterial reverse mutation test and the mammalian micronucleus test suggest that synthetic curcumin is not of mutagenic concern, and the results of the 14- and 90-day repeated oral dose studies, with a 90-day NOAEL of 1000 mg/kg bw/day (the highest dose tested), suggest that the compound is of no toxicological concern.

## Figures and Tables

**Table 1:** Mutagenicity assay: mean revertant frequency in the absence of metabolic activation system.

**Concentrations ** **(** **µ** **g/plate)**	**TA1537**		**TA1535**		**TA98**		**TA100**		**TA102**	
	**Mean ± SD**	**#**	**Mean ± SD**	**#**	**Mean ± SD**	**#**	**Mean ± SD**	**#**	**Mean ± SD**	**#**
Vehicle Control (0.0)	10.00 ± 3.61	NA	14.00 ± 1.00	NA	25.33 ± 1.53	NA	100.33 ± 14.01	NA	344.00 ± 14.42	NA

5.0	10.67 ± 0.58	1.07	20.00 ± 13.00	1.43	23.00 ± 6.24	0.91	104.00 ± 7.94	1.04	377.33 ± 26.63	1.10

16.0	9.67 ± 4.62	0.97	13.00 ± 4.36	0.93	21.00 ± 1.00	0.83	115.00 ± 3.00	1.15	370.67 ± 27.23	1.08

50.0	14.67 ± 3.06	1.47	10.67 ± 1.15	0.76	24.00 ± 5.29	0.95	118.00 ± 13.23	1.18	365.33 ± 43.14	1.06

160.0	11.67 ± 0.58	1.17	12.67 ± 3.06	0.91	23.67 ± 2.31	0.93	111.00 ± 10.15	1.11	305.33 ± 65.77	0.89

500.0	11.33 ± 4.04	1.13	12.67 ± 2.52	0.91	27.67 ± 4.73	1.09	98.00 ± 15.87	0.98	266.67 ± 24.44	0.78

1600.0	11.33 ± 3.06	1.13	11.67 ± 4.51	0.83	32.67 ± 16.86	1.42	96.00 ± 7.94	0.92	274.00 ± 57.65	0.80

9AA (50.0)	1043.33 ± 51.32	104.33	-	-	-	-	-	-		-

2NF (25.0)	-	-	-	-	1260.00 ± 65.57	49.74	-	-	-	-

SA (20.0)	-	-	1110.00 ± 105.36	79.29	-	-	1410.00 ± 36.06	14.05	-	-

Ametycin (0.25)	-	-	-	-	-	-	-	-	1626.67 ± 211.97	4.73

9AA, 9-aminoacridine; NA, not applicable; 2NF, 2-nitrofluorene; MMC, mitomycin C; SA, sodium azide; SD, standard deviation; VC, vehicle control; #, relative fold values as compared to VC.

*Note*. Vehicle is dimethyl sulfoxide; 9AA, 2NF, SA, and ametycine are positive controls.

**Table 2:** Mutagenicity assay: mean revertant frequency in the presence of metabolic activation system (5% v/v, S9).

**Concentration ** **(** **µ** **g/plate)**	**TA1537**		**TA1535**		**TA98**		**TA100**		**TA102**	
	**Mean ± SD**	**#**	**Mean ± SD**	**#**	**Mean ± SD**	**#**	**Mean ± SD**	**#**	**Mean ± SD**	**#**
Vehicle Control 0.0	13.33 ± 0.58	NA	14.00 ± 3.61	NA	32.00 ± 9.54	NA	107.67 ± 5.13	NA	289.33 ± 64.38	NA

5.0	13.67 ± 5.13	1.03	12.00 ± 1.00	0.86	22.00 ± 4.36	0.69	107.00 ± 6.56	0.99	302.00 ± 20.88	1.04

16.0	15.00 ± 0.00	1.13	11.67 ± 2.52	0.83	28.67 ± 6.81	0.90	114.67 ± 4.51	1.07	340.67 ± 37.22	1.18

50.0	14.00 ± 1.00	1.05	11.67 ± 2.89	0.83	23.67 ± 2.52	0.74	116.00 ± 35.09	1.08	362.67 ± 25.17	1.25

160.0	13.67 ± 2.08	1.03	13.33 ± 2.08	0.95	23.00 ± 4.36	0.72	121.00 ± 9.64	1.12	280.67 ± 33.49	0.97

500.0	11.67 ± 3.79	0.88	11.33 ± 1.53	0.81	24.00 ± 5.57	0.75	88.33 ± 6.66	0.82	353.33 ± 30.55	1.22

1600.0	11.00 ± 1.73	0.83	10.00 ± 3.00	0.71	36.33 ± 17.62	1.65	101.67 ± 22.19	0.95	322.67 ± 44.06	1.12

2AA (20.0)	1026.67 ± 140.12	77.02	1036.67 ± 130.51	74.05	1180.00 ± 60.00	36.88	1533.33 ± 135.03	14.24	1740.00 ± 87.18	6.01

2AA, 2-aminoanthracene; NA, not applicable; SD, standard deviation; #, relative fold values as compared to Vehicle Control.

*Note*. Vehicle is dimethyl sulfoxide; 2AA is the positive control.

**Table 3:** Summary data for mitotic index.

**Chromosome Aberration Assay – Short Term Exposure (Approximately 4 h)**							
**In the Absence of Metabolic Activation System**				**In the Presence of Metabolic Activation System**			
**Test Concentrations**	%** MI**	%	%** Relative**	**Test Concentrations**	%** MI**	%	%** Relative**
**(** **µ** **g/mL)**	** Mean ± SD**	**Relative MI**	**Reduction of MI**	** (** **µ** **g/mL)**	**Mean ± SD**	**Relative MI**	**Reduction of MI**
Vehicle Control 0.0	10.070 ± 0.69	NA	NA	Vehicle Control 0.0	8.733 ± 0.51	NA	NA

10.0	9.685 ± 0.69	96	4	6.3	7.296 ± 0.70	84	16

20.0	7.546 ± 0.21	75	25	12.5	5.594 ± 0.56	64	36

40.0	5.988 ± 1.13	59	41	25.0	4.595 ± 0.56	53	47

MMC 0.25	5.542 ± 0.22	55	45	CPA 12.5	5.050 ± 0.64	58	42

**Chromosome Aberration Assay – Continuous Exposure (Approximately 22 h)**							
**In the Absence of Metabolic Activation System**							
**Test Concentrations **		%** MI **		%		%** Relative **	
**(** **µ** **g/mL)**		**Mean ± SD**		**Relative MI**		**Reduction of MI**	

Vehicle Control 0.0		7.896 ± 0.56		NA		NA	

6.3		5.900 ± 0.42		75		25	

12.5		5.290 ± 0.57		67		33	

25.0		3.882 ± 0.55		49		51	

CPA 12.5		4.389 ± 0.27		56		44	

CPA, cyclophosphamide, positive control; MI, mitotic index; % MI, number of mitotic cells x 100/total number of cells scored; MMC, mitomycin C, positive control; NA, not applicable; SD, standard deviation; VC = vehicle control (dimethyl sulfoxide).

**Table 4:** Summary data for chromosome aberrations.

**Short Term Exposure**								**Continuous Exposure**			
**In the Absence of Metabolic Activation System**				**In the Presence of Metabolic Activation System**				**In Absence of Metabolic Activation System**			
**Concentration (** **µ** **g/mL) **	%** Aberrated Cells (Structural)**		% **Numerical Aberration Mean ± SD**	**Concentration (** **µ** **g/mL) **	%** Aberrated Cells (Structural)**		% **Numerical Aberration Mean ± SD**	**Concentration (** **µ** **g/mL)**	%** Aberrated Cells (Structural)**		% **Numerical Aberration Mean±SD**
	**Including Gap #** **Mean ± SD**	**Excluding Gap #** **Mean ± SD**			**Including Gap #** **Mean ± SD**	**Excluding Gap #** **Mean ± SD**			**Including Gap #** **Mean ± SD**	**Excluding Gap #** **Mean ± SD**	
Vehicle Control 0.0	1.00 ± 0.47	0.67 ± 0.94	0.40 ± 0.57	Vehicle Control 0.0	1.33 ± 0.00	1.00 ± 0.47	0.00 ± 0.00	Vehicle Control 0.0	1.34 ± 0.94	1.34 ± 0.94	0.00 ± 0.00

10.0	1.33 ± 0.00	1.00 ± 0.47	0.40 ± 0.00	6.3	0.67 ± 0.00	0.34 ± 0.47	1.00 ± 0.28	6.3	1.67 ± 0.47	1.33 ± 0.00	0.20 ± 0.28

20.0	3.34 ± 1.89	3.00 ± 1.41	0.60 ± 0.28	12.5	2.00 ± 0.95	1.67 ± 0.47	0.40 ± 0.00	12.5	1.33 ± 0.00	1.33 ± 0.00	0.20 ± 0.28

40.0	1.67 ± 0.47	1.67 ± 0.47	0.40 ± 0.00	25.0	4.33*∗* ± 1.41	4.33*∗* ± 1.41	0.60 ± 0.28	25.0	2.33 ± 1.41	2.00 ± 0.95	0.20 ± 0.28

MMC 0.25	12.33*∗∗* ± 0.47	12.33*∗∗* ± 0.47	0.00 ± 0.00	CPA 12.5	17.67*∗∗* ± 3.30	17.33*∗∗* ± 2.83	0.20 ±0.28	MMC 0.25	11.33*∗∗* ± 0.00	11.33*∗∗* ± 0.00	0.00 ± 0.00

Historical Vehicle Control^*♮*^	0.89–1.61	0.40–0.94	NA	Historical Vehicle Control^*♮*^	0.82–1.39	0.46–1.04	NA	Historical Vehicle Control^*♮*^	0.76–1.61	0.39–1.07	NA

CPA, cyclophosphamide, positive control; MMC, mitomycin C, positive control; SD, standard deviation; VC, vehicle control (dimethyl sulfoxide); *∗*, significantly increased at 5 % in two sided Fisher's Exact Test; *∗∗*, significantly increased at 1 % in two sided Fisher's Exact Test; #, number of aberrant cells; ^*♮*^, numbers reported are the 95% confidence interval; NA, not applicable.

**Table 5:** Summary of results for the mouse micronucleus test.

**Groups** n=5	**Polychromatic Erythrocytes/** **Total Erythrocytes** **Mean ± SD**		%** MN – PCE** **(mean & SD)**		**Total MNPCE**	
	**Male**	**Female**	**Male**	**Female**	**Male**	**Female**
Vehicle Control	0.515 ± 0.03	0.517 ± 0.02	0.030 ± 0.02	0.040 ± 0.01	06	08

500 mg/kg bw	0.517 ± 0.01	0.524 ± 0.01	0.025 ± 0.02	0.035 ± 0.02	05	07

1000 mg/kg bw	0.520 ± 0.01	0.520 ± 0.01	0.035 ± 0.02	0.040 ± 0.02	07	08

2000 mg/kg bw	0.521 ± 0.01	0.521 ± 0.00	0.035 ± 0.02	0.035 ± 0.01	07	07

Positive control 30 mg/kg bw	0.481 ± 0.02*∗*	0.477 ± 0.01*∗∗*	1.042 *∗∗* ± 0.17	1.053 *∗∗* ± 0.14	209	211

Historical Vehicle Control Data	0.545 ± 0.063	0.567 ± 0.056	0.045 ± 0.051	0.072 ± 0.065	NA	NA

*Note*. Vehicle control, 0.5 % w/v carboxymethylcellulose; SD, standard deviation; positive control, CP-cyclophosphamide monohydrate; test item, curcumin in mg/kg bw.

**Table 6:** Summary of detailed clinical examination*∗*.

**Week**	**Group / Dose (mg/kg bw/day)**				
	**Vehicle 0**	**250**	**500**	**1000**	**Ref Item 1000**
**Males**					
Pre-Dose	NAD / 20	NAD / 20	NAD / 20	NAD / 20	NAD

1	NAD / 20	NAD / 20	NAD / 20	NAD / 20	NAD

2	NAD / 20	NAD / 20	NAD / 20	NAD / 20	NAD

3	NAD / 20	NAD / 20	NAD / 20	NAD / 12, Tail is yellowish in colour / 8	Tail is yellowish in colour/ 20

4	NAD / 20	NAD / 20	NAD / 20	NAD / 4, Tail is yellowish in colour/ 16	Tail is yellowish in colour/ 20

5	NAD / 20	NAD / 20	NAD / 20	Tail is yellowish in colour/ 20	Tail is yellowish in colour/ 20

6	NAD / 20	NAD / 20	NAD / 20	Tail is yellowish in colour/ 20	Tail is yellowish in colour/ 20

7	NAD / 20	NAD / 20	NAD / 20	Fur is slight yellowish in colour/20, Tail is yellowish in colour/ 20	Fur is slight yellowish in colour/20, Tail is yellowish in colour/ 20

8	NAD / 20	NAD / 20	NAD / 20	Fur is slight yellowish in colour/20, Tail is yellowish in colour/ 20	Fur is slight yellowish in colour/20, Tail is yellowish in colour/ 20

9	NAD / 20	NAD / 20	NAD / 20	Fur is slight yellowish in colour/20, Tail is yellowish in colour/ 20	Fur is slight yellowish in colour/20, Tail is yellowish in colour/ 20

10	NAD / 20	NAD / 20	NAD / 20	Fur is slight yellowish in colour/20, Tail is yellowish in colour/ 20	Fur is slight yellowish in colour/20, Tail is yellowish in colour/ 20

11	NAD / 20	NAD / 20	NAD / 20	Fur is slight yellowish in colour/20, Tail is yellowish in colour/ 20	Fur is slight yellowish in colour/20, Tail is yellowish in colour/ 20

12	NAD / 20	NAD / 20	NAD / 20	Fur is slight yellowish in colour/20, Tail is yellowish in colour/ 20	Fur is slight yellowish in colour/20, Tail is yellowish in colour/ 20

13	NAD / 20	NAD / 20	NAD / 20	Fur is slight yellowish in colour/20, Tail is yellowish in colour/ 20	Fur is slight yellowish in colour/20, Tail is yellowish in colour/ 20

**Females**					
Pre-Dose	NAD / 20	NAD / 20	NAD / 20	NAD / 20	NAD / 20

1	NAD / 20	NAD / 20	NAD / 20	NAD / 20	NAD / 20

2	NAD / 20	NAD / 20	NAD / 20	NAD / 20	NAD / 20

3	NAD / 20	NAD / 20	NAD / 20	NAD / 11, Tail is yellowish in colour/ 9	Tail is yellowish in colour/ 20

4	NAD / 20	NAD / 20	NAD / 20	NAD / 3, Tail is yellowish in colour/ 17	Tail is yellowish in colour/ 20

5	NAD / 20	NAD / 20	NAD / 20	Tail is yellowish in colour/ 20	Tail is yellowish in colour/ 20

6	NAD / 20	NAD / 20	NAD / 20	Tail is yellowish in colour/ 20	Tail is yellowish in colour/ 20

7	NAD / 20	NAD / 20	NAD / 20	Tail is yellowish in colour/ 20	Fur is slight yellowish in colour / 6, Tail is yellowish in colour/ 20

8	NAD / 20	NAD / 20	NAD / 20	Tail is yellowish in colour/ 20	Fur is slight yellowish in colour/ 8, Tail is yellowish in colour/ 20

9	NAD / 20	NAD / 20	NAD / 20	Tail is yellowish in colour/ 20	Fur is slight yellowish in colour / 8, Tail is yellowish in colour/ 20

10	NAD / 20	NAD / 20	NAD / 20	Tail is yellowish in colour/ 20	Fur is slight yellowish in colour / 9, Tail is yellowish in colour/ 20

11	NAD / 20	NAD / 20	NAD / 20	Tail is yellowish in colour/ 20	Fur is slight yellowish in colour / 9, Tail is yellowish in colour/ 20

12	NAD / 20	NAD / 20	NAD / 20	Tail is yellowish in colour/ 20	Fur is slight yellowish in colour / 9, Tail is yellowish in colour/ 20

13	NAD / 20	NAD / 20	NAD / 20	Tail is yellowish in colour/ 20	Fur is slight yellowish in colour / 9, Tail is yellowish in colour/ 20

*∗*: results shown as clinical signs/number of animals showing particular clinical signs.

NAD: no abnormality detected; 20: number of animals per group.

**Table 7:** Mean body weights for the 90-day study on curcumin.

**Group **	**Day / Body Weight (g)**														
n=20 mg/kg bw/day		**1**	**8**	**15**	**22**	**29**	**36**	**43**	**50**	**57**	**64**	**71**	**78**	**85**	**90**
	**Males**														
**Vehicle**	Mean	174.28	212.83	243.56	271.50	299.19	318.98	337.42	351.65	364.72	376.89	384.98	392.33	397.09	400.54
**0**	SD	11.51	17.19	20.13	22.94	25.01	24.35	29.40	31.89	33.48	34.64	35.06	35.57	36.63	37.18

**250**	Mean	177.16	214.00	246.07	271.88	296.26	315.99	333.32	344.86	355.03	365.52	371.93	376.73	381.17	383.93
	SD	13.74	13.18	14.32	16.72	20.18	22.36	25.21	26.03	26.39	29.27	31.54	32.90	33.00	34.52

**500**	Mean	178.57	217.82	254.98	286.03	313.89	335.72	356.92	369.90	382.61	393.92	401.14	406.57	415.12	417.18
	SD	13.42	13.70	14.47	14.65	17.14	18.84	21.18	23.29	25.48	27.46	28.50	28.04	29.20	29.09

**1000**	Mean	173.03	213.96	246.54	272.97	301.45	323.37	340.95	352.78	366.35	376.83	382.72	389.27	397.12	400.97
	SD	12.08	12.73	12.13	14.37	16.96	19.66	22.28	24.26	27.01	26.05	24.46	25.49	25.65	26.01

**Ref Item**	Mean	171.34	209.30	240.81	265.27	291.83	309.92	326.03	339.20	350.40	361.16	367.49	374.05	378.81	383.67
**1000**	SD	13.75	18.47	20.28	22.27	24.62	25.15	25.43	26.11	27.44	30.72	31.23	31.68	34.20	33.83

	**Females**														
**Vehicle**	Mean	142.22	162.16	177.00	187.93	199.51	206.83	214.65	219.22	223.98	227.10	229.34	232.61	234.13	234.84
**0**	SD	12.84	12.25	14.21	14.82	14.53	14.68	15.43	15.23	16.27	16.33	15.79	16.18	16.85	16.48

**250**	Mean	141.57	163.45	180.25	191.80	200.57	208.29	217.53	219.72	222.27	225.41	229.64	230.38	232.30	232.85
	SD	10.72	10.58	11.89	11.89	12.42	11.83	11.68	12.18	13.51	13.39	13.79	14.63	15.35	15.15

**500**	Mean	142.24	161.19	177.56	187.87	199.22	204.19	212.63	215.23	221.07	223.30	226.21	227.48	229.99	231.08
	SD	10.61	10.44	12.33	12.66	13.83	14.22	14.66	14.63	15.63	15.81	16.82	16.71	17.05	17.21

**1000**	Mean	142.48	161.92	175.93	187.59	202.90	212.25	218.30	222.86	228.31	231.99	233.25	237.91	239.97	241.14
	SD	11.95	12.56	16.62	15.34	16.10	16.75	18.76	18.99	19.42	19.02	19.97	20.60	21.60	21.89

**Ref **	Mean	142.97	161.96	175.80	188.52	201.32	208.54	214.51	219.45	223.55	227.50	230.28	233.14	234.30	235.08
**Item ** **1000**	SD	13.34	14.44	14.04	15.83	17.70	18.16	18.35	19.42	20.53	20.19	18.94	18.22	19.05	18.82

n, number of animals per group; SD, standard deviation.

**Table 8:** Summary of feed consumption for the 90-day study on curcumin.

**Group**		Feed Consumption (g) /day/animal													
n=20 mg/kg bw/day	**Days**	**1 - 2**	**8-9**	**15-16**	**22-23**	**29-30**	**36-37**	**43-44**	**50-51**	**57-58**	**64-65**	**71-72**	**78-79**	**85-86**	**89-90**
**Males**															

**Vehicle **	Mean	20.55	21.86	21.96	21.89	23.15	22.40	21.58	21.49	21.07	21.46	21.89	22.36	22.60	21.17
**0**	SD	2.25	1.47	2.55	2.07	3.46	1.43	1.45	1.37	1.48	1.59	1.51	1.09	1.64	0.68

**250**	Mean	19.92	22.19	21.57	21.03	23.97	21.28	21.67	19.56*∗*-	22.19	21.30	20.42	21.03	21.20	20.88
	SD	1.92	1.45	1.52	1.20	3.49	1.32	1.39	1.01	0.65	1.35	1.50	1.08	1.13	0.95

**500**	Mean	40.80*∗*+	23.70	22.80	21.50	26.28*∗*+	22.37	22.33	21.05	21.99	21.75	21.30	21.18	21.63	21.14
	SD	2.52	2.42	1.28	0.96	4.32	1.04	0.85	0.91	0.96	1.67	1.00	1.71	1.37	0.70

** 1000**	Mean	19.99	23.71*∗*+ ↑	22.68	23.24↑	24.06↑	23.47↑	22.72↑	21.64	20.22	21.35↑	21.59↑	21.58	22.48	21.13
	SD	1.17	2.44	1.24	1.26	3.36	1.33	1.44	1.13	1.37	1.55	1.25	1.06	1.28	1.23

**Ref Item**	Mean	17.30*∗*-	21.27	21.54	21.51	20.76	21.16	20.19	21.34	19.77	19.83	19.91*∗*-	20.80*∗*-	21.27	21.85
**1000**	SD	3.80	1.96	1.82	1.42	1.56	1.37	1.17	2.06	1.75	1.39	1.74	1.12	1.60	0.76

**Females**															

**Vehicle**	Mean	15.07	15.04	16.23	16.18	16.66	17.89	14.65	16.82	16.42	17.08	16.23	16.08	14.81	16.59
**0**	SD	2.54	1.08	1.28	1.30	1.18	0.55	0.79	1.31	1.02	0.94	0.79	0.66	0.44	1.53

**250**	Mean	15.51	13.92	16.71	16.45	17.75	14.62*∗∗*-	15.75*∗*+	17.10	15.51	16.28	15.23	16.58	15.36	15.92
	SD	1.29	1.15	1.07	1.72	1.40	1.64	0.97	1.81	2.02	1.96	2.18	1.13	1.75	1.27

**500**	Mean	12.76*∗*-	14.03	15.85	15.98	16.46	16.32*∗*-	15.17	17.44	14.43*∗∗*-	15.38*∗*-	16.03	16.26	14.75	15.26
	SD	1.05	1.77	1.47	1.77	1.45	1.92	1.75	0.46	1.11	1.46	1.57	1.08	1.03	1.23

**1000**	Mean	15.46	15.11	16.31	16.61	16.92	18.80	16.21*∗∗*+	17.18	16.45	17.19	16.81	16.54	16.45*∗∗*+	16.31
	SD	1.30	1.85	2.08	1.90	1.81	1.17	1.08	1.37	0.71	0.76	0.98	1.28	1.93	1.33

**Ref Item**	Mean	15.75	15.51	15.47	16.64	17.17	18.49	16.02	16.12	17.31	16.76	16.37	16.31	16.01	16.22
**1000**	SD	2.26	2.00	2.97	2.64	2.60	2.43	2.94	0.64	2.39	1.23	1.13	1.19	2.34	1.33

n= number of animals,  ^**∗**+/**∗**-^**=** statistically significant increase/decrease as compared to vehicle control (p<0.05),  ^**∗∗**+/**∗∗**-^**=** statistically significant increase/decrease as compared to vehicle control (p<0.01), and ↑/↓ = statistically significant increase/decrease as compared to the reference item group (p<0.05); Ref, reference.

**Table 9:** Summary of significant Hematology measures in the 90-day study on curcumin.

**Group **		**RBC**	**HGB**	**MCV**	**MCH**	**MCHC**	**RDW**	**Retic**	**MPV**	**WBC**	**Neu**	**Lymp**	**Mono**	**Mono**	**Plt**
n=20 mg/kg bw/day		(10^6^ cells/*µ*L)	(g/dL)	(fL)	(pg)	(g/dL)	(%)	(x10^9^ cells/L)	(fL)	(10^3^ cells/*µ*L)	(10^3^ cells/ *µ*L)	(10^3^ cells/*µ*L)	(10^3^ cells/*µ*L)	(%)	(x10^3^ cells/*µ*L)
**Males**															

**Vehicle**	Mean	9.12	15.65	54.02	17.17	31.80	12.55	110.20	9.01	7.07	1.74	5.00	0.17	2.42	856.95
**0**	SD	0.26	0.41	1.74	0.70	0.56	0.63	20.54	0.37	1.39	0.78	1.02	0.08	0.71	114.07

**250**	Mean	8.84*∗∗*-	15.50	55.18	17.55	31.81	12.95*∗∗*+	116.79	8.76*∗*-	6.35	1.40	4.67	0.13*∗*-	1.94*∗*-	831.50
	SD	0.33	0.49	1.28	0.49	0.71	0.35	16.55	0.61	1.15	0.50	0.93	0.05	0.56	113.52

**500**	Mean	8.76*∗∗*-	15.43*∗∗*-	56.37*∗∗*+	17.63	31.27*∗*-	13.05*∗∗*+	104.27	8.76*∗*-	5.37*∗∗*-	1.22*∗∗*-	3.91*∗∗*-	0.12*∗∗*-	2.42	853.30
	SD	0.71	1.22	2.15	0.78	0.58	0.58	32.83	0.21	1.12	0.44	0.79	0.09	2.23	87.57

**1000**	Mean	8.67*∗∗*-	15.43*∗*-	55.57*∗*+	17.84*∗*+	32.09	12.57	111.90	9.56*∗∗*+	6.10*∗*-↑	1.19*∗*-	4.65↑↑	0.10*∗∗*-	1.60*∗∗*-↓	812.55
	SD	0.47	0.39	2.40	0.80	0.54	0.57	21.42	0.55	1.20	0.42	0.96	0.03	0.30	91.69

**Ref Item**	Mean	8.75*∗∗*-	15.71*∗∗*+	55.69*∗*+	17.97*∗∗*+	32.26	12.65	98.60	9.34*∗∗*+	5.35*∗∗*-	1.18*∗∗*-	3.93*∗∗*-	0.10*∗∗*-	1.87*∗*-	816.00
**1000**	SD	0.40	0.52	1.90	0.69	0.62	0.34	25.64	0.38	0.76	0.37	0.68	0.03	0.42	107.22
**Historical Range**		7.67–9.45	13.00–16.00	46.00–54.80	14.30–17.90	30.60–34.60	11.90–14.30	40.70–164.30	6.7–11.6	3.72–10.81	0.84–2.94	2.45–8.85	0.07–0.27	1.30–2.70	650–1058

**Females**															

**0**	Mean	8.15	14.92	57.17	18.30	32.04	11.56	116.71	7.63	4.96	1.20	3.53	0.11	2.05	855.65
	SD	0.29	0.56	1.75	0.62	0.48	0.44	27.68	0.43	1.99	0.92	1.09	0.08	0.81	91.24

**250**	Mean	8.09	15.39*∗∗*+	57.94	19.04*∗∗*+	32.86*∗∗*+	11.13*∗*-	145.26*∗*+	8.59*∗∗*+	5.11	1.02	3.84	0.10	1.94	870.45
	SD	0.29	0.52	1.76	0.59	0.49	0.39	35.77	0.63	1.05	0.39	0.95	0.03	0.39	103.34

**500**	Mean	8.11	15.08	56.63	18.61	32.84*∗∗*+	11.25	116.62	8.62*∗∗*+	3.58*∗∗*-	0.68*∗∗*-	2.73*∗∗*-	0.06*∗*-	1.67	886.75
	SD	0.29	0.31	1.31	0.46	0.68	0.46	27.41	0.46	0.89	0.24	0.68	0.02	0.49	88.98

**1000**	Mean	8.22	15.13	57.17	18.44	32.26	11.38	98.44↓	7.47	4.29	0.97	3.11	0.08	1.98	893.45
	SD	0.35	0.49	1.55	0.59	0.65	0.44	28.90	0.35	1.11	0.36	1.00	0.03	0.59	109.44

**Ref item **	Mean	8.01	14.92	57.36	18.65	32.51	11.56	117.96	7.41	4.54	1.05	3.24	0.09	1.90	863.10
**1000**	SD	0.31	0.48	2.11	0.73	0.78	0.59	31.28	0.61	1.08	0.43	0.90	0.04	0.65	90.93

**Historical Range**		6.64–8.87	12.00–15.20	49.70–58.20	16.10–18.60	31.10–35.40	10.60–14.30	72.40–221.30	6.5–11.5	3.14–10.03	0.60–2.24	1.24–7.07	0.05–0.25	1.10–3.60	560–1047

Key: n= No. of animals,  ^**∗**+/**∗**-^**=** Statistically significant increase/decrease as compared to vehicle control (p<0.05),  ^**∗∗**+/**∗∗**-^**=** Statistically significant increase/decrease as compared to vehicle control (p<0.01), ↑/↓= Statistically significant increase/decrease as compared to the reference item group (p<0.05), ↑↑ = Statistically significant increase as compared to the reference item group (p<0.01); Ref, reference.

Abbreviations: HGB, hemoglobin; Lymp, lymphocytes; MCH, mean corpuscular hemoglobin; MCHC, mean corpuscular hemoglobin concentration; MCV, mean corpuscular volume; Mono, monocytes; MPV, mean platelet volume; Neu, neutrophils; Plt, platelets; RBC, red blood cells; RDW, red cell distribution width; Retic, reticulocytes; WBC, white blood cells.

**Table 10:** Summary of significant clinical chemistry results for the 90-day study on curcumin.

**Group**		**Glu**	**T Prot**	**Alb**	**Glob**	**A/G R**	**Tg**	**TC**	**AST**	**ALT**	**ALP**	**Tbil**	**BUN**	**Crea**	**Na**	**K**	**Cl**	**Ca**	P
n=20 mg/kg bw/day		(mg/dL)	(g/dL)	(g/dL)	(g/dL)	-	(mg/dL)	(mg/dL)	(U/L)	U/L)	(U/L)	(mg/dL)	(mg/dL)	(mg/dL)	(mmol/L)	(mmol/L)	(mmol/L)	(mmol/L)	(mg/dL)
	**Males**																		

**Vehicle**	Mean	99.45	6.22	3.38	2.83	1.20	79.70	69.60	89.20	41.40	91.90	0.20	15.95	0.43	150.10	4.61	110.25	10.46	6.00
**0**	SD	12.82	0.20	0.16	0.15	0.09	29.79	11.54	8.28	7.11	13.53	0.04	2.33	0.04	1.65	0.22	1.62	0.28	0.55

** 250**	Mean	89.45	6.14	3.39	2.74	1.24	60.35	55.75*∗∗*-	101.50*∗∗*+	44.50	85.05	0.17	17.00	0.44	151.75*∗∗*+	4.64	110.15	10.46	6.20
	SD	20.84	0.25	0.21	0.14	0.10	12.25	12.84	15.04	10.76	11.81	0.04	2.70	0.05	1.68	0.46	1.39	0.31	0.54

**500**	Mean	86.90*∗∗*-	6.25	3.47	2.78	1.25	80.35	62.30	87.80	42.85	89.55	0.20	17.85*∗*+	0.43	150.75	4.39*∗∗*-	109.90	10.72*∗∗*+	6.27
	SD	12.60	0.21	0.21	0.12	0.11	41.86	15.51	11.37	8.99	16.13	0.04	1.70	0.05	1.33	0.22	1.71	0.27	0.61

** 1000**	Mean	87.85*∗∗*-	6.14	3.41	2.73*∗*-	1.25	77.60	72.60	98.45	43.45	92.05	0.21	16.11	0.41	149.90	4.44*∗*-	110.60↑↑	10.39	6.57*∗∗*+↑↑
	SD	11.82	0.16	0.12	0.11	0.07	23.79	11.57	17.61	9.83	15.97	0.05	2.17	0.03	1.45	0.21	1.50	0.25	0.48

**Ref Item**	Mean	97.40	6.25	3.47	2.78	1.25	74.85	74.45	91.50	43.25	97.50	0.20	15.46	0.43	149.00	4.53	109.20	10.49	5.96
**1000**	SD	27.52	0.20	0.15	0.12	0.08	26.62	16.22	13.65	8.34	15.57	0.05	1.74	0.06	1.72	0.28	1.67	0.20	0.43

**Historical Ranges**		65–115	5.84–6.71	2.92–3.57	2.76–3.40	0.91–1.23	43–125	56–92	91–217	33–131	68–170	0.50–0.89	12.60–25.10	0.40–0.79	140–149	3.80–5.50	100–132	9.60–10.54	5.29–7.65

	**Females**																		

**Vehicle**	Mean	74.50	6.62	3.99	2.63	1.52	68.85	67.50	119.40	31.80	48.55	0.24	20.13	0.46	149.10	4.15	107.55	10.72	5.04
**0**	SD	12.03	0.27	0.30	0.13	0.16	21.97	13.99	20.74	4.44	11.07	0.05	3.27	0.05	1.45	0.27	1.85	0.24	0.58

**250**	Mean	84.10*∗*+	6.56	3.94	2.62	1.51	51.20*∗∗*-	51.15*∗∗*-	86.65*∗∗*-	34.95	42.75	0.22	22.02	0.45	150.05*∗*+	4.14	110.00*∗∗*+	10.89	5.06
	SD	9.59	0.25	0.31	0.13	0.17	13.00	13.47	13.35	8.08	7.76	0.06	3.65	0.04	1.47	0.27	1.03	0.23	0.47

**500**	Mean	85.05*∗*+	6.42	3.78	2.64	1.43	41.30*∗∗*-	54.30*∗∗*-	81.80*∗∗*-	30.45	43.40	0.21	22.00	0.47	151.60*∗∗*+	4.09	112.40*∗∗*+	10.91	5.09
	SD	12.05	0.33	0.31	0.09	0.12	9.69	15.65	15.42	3.46	8.93	0.04	3.59	0.05	1.57	0.27	2.23	0.33	0.48

**1000**	Mean	73.45	6.53	3.87	2.65	1.47	48.90*∗∗*-	66.75	102.30*∗∗*-	34.55	47.55	0.24	20.31	0.45	147.80*∗∗*-↑↑	4.27	107.85↑	10.76	5.52
	SD	9.69	0.30	0.33	0.15	0.18	9.16	14.14	43.00	13.44	14.41	0.06	2.62	0.07	1.51	0.36	2.32	0.32	1.41

**Ref Item**	Mean	78.65	6.71	4.11	2.60	1.59	53.90*∗*-	62.85	96.40*∗∗*-	33.50	43.80	0.23	22.41	0.46	145.65*∗∗*-	4.37	105.90*∗*-	10.78	5.49
**1000**	SD	12.54	0.30	0.43	0.15	0.27	15.00	18.87	29.69	12.54	7.51	0.05	2.53	0.07	2.62	1.14	2.53	0.31	0.85

**Historical Ranges**		60–99	5.45–7.28	2.85–4.03	2.77–3.38	0.90–1.42	30–80	45–86	68–177	19–50	23–68	0.55–0.81	14.50–26.80	0.40–0.85	142–149	3.20–4.80	100–132	9.90–11.54	4.61–8.00

N= number of animals,  ^**∗**+/**∗**-^**=** statistically significant increase/decrease as compared to vehicle control (p<0.05),  ^**∗∗**+/**∗∗**-^**=** statistically significant increase/decrease as compared to vehicle control (p<0.01), ↑= statistically significant increase/decrease as compared to the reference item group (p<0.05), and ↑↑= statistically significant increase/decrease as compared to the reference item group (p<0.01)

A/G R, albumin/globulin ratio; Alb, albumin; ALP, alkaline phosphatase; ALT, alanine aminotransferase; AST, aspartate aminotransferase; BUN, blood urea nitrogen; Ca, calcium; Cl, chloride; Crea, creatinine; Glob, globulin; Glu, glucose, K, potassium; P, phosphorus; Na, sodium; Tbil, total bilirubin; TC, total cholesterol; Tg, triglycerides; and T Prot, total protein.

**Table 11:** Summary of coagulation parameters for the 90-day study on curcumin.

**Group ** **n=20**		**PT**	**APTT**	**Fibrinogen**
**mg/kg bw/day**		(Sec)	(Sec)	(mg/dl)
**Males**				

**Vehicle ** ** 0**	Mean	17.42	16.53	431.05
	SD	3.34	5.86	117.12

** 250**	Mean	17.29	19.03	336.03*∗∗*-
	SD	5.08	5.16	70.20

** 500**	Mean	14.84*∗∗*-	14.67	407.63
	SD	1.99	3.13	55.83

** 1000**	Mean	14.84*∗∗*-↓	13.73	355.00*∗∗*-
	SD	1.64	2.37	62.36

**Reference Item ** **1000**	Mean	16.30	13.48*∗*-	392.72
	SD	1.91	4.35	57.90

**Historical Range**		14.90–21.00	10.00–19.60	145.30–985.40

**Females**				

**Vehicle** **0**	Mean	15.08	16.32	268.18
	SD	0.75	2.83	63.57

**250**	Mean	18.45*∗∗*+	14.47	264.64
	SD	1.34	3.19	188.79

**500**	Mean	17.55*∗∗*+	12.72*∗∗*-	241.01
	SD	2.51	2.38	50.99

**1000**	Mean	14.83↓	17.97	324.30
	SD	0.66	3.50	263.26

**Reference Item ** **1000**	Mean	19.20*∗∗*+	16.87	246.29
	SD	1.69	3.71	44.00

**Historical Range**		14.50–21.90	7.40–21.00	243.50–758.10

N= number of animals,  ^**∗**+/**∗**-^**=** statistically significant increase/decrease as compared to vehicle control (p<0.05),  ^**∗∗**+/**∗∗**-^**=** statistically significant increase/decrease as compared to vehicle control (p<0.01), and ↓ = statistically significant decrease as compared to the reference item group (p<0.05).

APTT, activated partial thromboplastin time; PT, prothrombin time.

**Table 12:** Summary of urinalysis parameters.

**Parameters**	**Group** mg/kg bw/day				
	**Vehicle** **0**	**250**	**500**	**1000**	**Ref Item** **1000**
	**Males**				

**Colour**					
Light Yellow	20/20	16/20	12/20	19/20	20/20
Yellow	-	4/20	1/20	1/20	-
Other	-	-	7/20	-	-

**Appearance**					
Clear	20/20	20/20	20/20	20/20	20/20

**Glucose**					
Negative	20/20	20/20	19/20	20/20	20/20
100	-	-	1/20	-	-

**Protein**					
Negative	18/20	4/20	2/20	18/20	20/20
10	2/20	-	3/20	1/20	-
30	-	1/20	6/20	1/20	-
100	-	9/20	7/20	-	-
300	-	3/20	1/20	-	-
1000	-	3/20	1/20	-	-

**Ketones Bodies**					
Negative	15/20	19/20	15/20	16/20	13/20
5	2/20	1/20	1/20	2/20	5/20
10	3/20	-	4/20	2/20	2/20

**Bilirubin**					
Negative	20/20	19/20	18/20	20/20	20/20
0.5	-	1/20	1/20	-	-
3.0	-	-	1/20	-	-

**Urobilinogen**					
Normal	20/20	20/20	17/20	20/20	20/20
1.0	-	-	2/20	-	-
12.0	-	-	1/20	-	-

**Nitrite**					
Negative	7/20	2/20	11/20	12/20	10/20
Positive	13/20	18/20	9/20	8/20	10/20

**Specific Gravity**					
1.005	4/20	8/20	4/20	3/20	4/20
1.010	10/20	10/20	14/20	14/20	13/20
1.015	6/20	2/20	2/20	3/20	3/20

**pH**					
6.5	1/20	-	-	-	-
7.0	2/20	-	1/20	-	3/20
7.5	5/20	8/20	3/20	10/20	7/20
8.0	6/20	6/20	11/20	4/20	5/20
8.5	1/20	4/20	3/20	3/20	4/20
9.0	5/20	2/20	2/20	3/20	1/20

**Occult Blood**					
Negative	20/20	20/20	17/20	19/20	19/20
5	-	-	3/20	-	1/20
10	-	-	-	1/20	-

**Leucocytes**					
Negative	20/20	20/20	20/20	19/20	20/20
10	-	-	-	1/20	-
**Microscopy**					
Epithelial Cell					
0	3/20	3/20	3/20	3/20	1/20
1+	14/20	12/20	8/20	13/20	14/20
2+	3/20	5/20	8/20	3/20	3/20
3+	-	-	1/20	1/20	2/20
Casts/Crystals					
1+	6/20	4/20	6/20	7/20	11/20
2+	1/20	12/20	7/20	4/20	4/20
3+	4/20	4/20	7/20	2/20	1/20
4+	9/20	-	-	7/20	4/20
Abnormal Cells					
NIL	20/20	20/20	20/20	20/20	20/20

	**Females**				

**Colour**					
Light Yellow	20/20	20/20	20/20	18/20	20/20
Yellow	-	-	-	2/20	-

**Appearance**					
Clear	20/20	20/20	20/20	20/20	20/20

**Glucose**					
Negative	20/20	20/20	20/20	20/20	20/20

**Protein**					
Negative	14/20	12/20	19/20	12/20	16/20
10	2/20	3/20	1/20	5/20	2/20
30	-	1/20	-	1/20	2/20
100	2/20	4/20	-	2/20	-
1000	2/20	-	-	-	-

**Ketones Bodies**					
Negative	20/20	20/20	20/20	20/20	20/20

**Bilirubin**					
Negative	20/20	20/20	20/20	20/20	20/20

**Urobilinogen**					
Normal	20/20	20/20	20/20	20/20	20/20

**Nitrite**					
Negative	4/20	5/20	5/20	6/20	18/20
Positive	16/20	15/20	15/20	14/20	2/20
Specific Gravity					
1.005	3/20	2/20	2/20	4/20	3/20
1.010	8/20	16/20	11/20	11/20	15/20
1.015	9/20	2/20	7/20	5/20	2/20

**pH**					
5.5	1/20	-	-	-	-
6.0	1/20	-	-	-	-
6.5	3/20	-	-	1/20	-
7.0	2/20	1/20	3/20	2/20	-
7.5	2/20	4/20	3/20	3/20	5/20
8.0	3/20	9/20	7/20	6/20	7/20
8.5	3/20	4/20	2/20	5/20	7/20
9.0	5/20	2/20	5/20	3/20	1/20

**Occult Blood**					
Negative	19/20	18/20	20/20	14/20	17/20
5	1/20	-	-	2/20	2/20
10	-	-	-	2/20	-
50	-	1/20	-	2/20	1/20
250	-	1/20	-	-	-

**Leucocytes**					
Negative	17/20	20/20	20/20	20/20	20/20

**Microscopy**					
Epithelial Cell					
0	1/20	-	-	4/20	2/20
1+	12/20	14/20	14/20	7/20	9/20
2+	7/20	6/20	6/20	8/20	6/20
3+	-	-	-	1/20	3/20
Casts/Crystals					
1+	5/20	5/20	4/20	5/20	3/20
2+	9/20	11/20	12/20	12/20	9/20
3+	6/20	4/20	4/20	3/20	8/20
Abnormal Cells					
NIL	20/20	20/20	20/20	20/20	20/20

**Table 13:** Summary of absolute organ weights for 90-day study on curcumin.

**Group**		**Absolute Organ Weights (g)**							
n=20 mg/kg bw/d		**Adrenal glands**	**Heart**	**Brain**	**Liver**	**Kidneys**	**Spleen**	**Testes/Ovaries**	**Epididymides/Uterus**
**Males**									

**Vehicle **	Mean	0.056	1.028	2.104	10.577	2.293	0.665	3.661	1.451
**0**	SD	0.007	0.070	0.119	1.290	0.252	0.082	0.366	0.141

** 250**	Mean	0.059	1.028	2.174	10.378	2.332	0.614	3.776	1.497
	SD	0.007	0.080	0.090	1.118	0.247	0.104	0.218	0.103

** 500**	Mean	0.062	1.101*∗*+	2.222*∗*+	11.452	2.605*∗∗*+	0.723	3.898*∗*+	1.546*∗*+
	SD	0.007	0.097	0.124	0.874	0.578	0.140	0.492	0.134

** 1000**	Mean	0.056	1.049	2.126	11.217	2.398	0.665	3.736	1.516
	SD	0.011	0.079	0.161	1.263	0.176	0.106	0.416	0.117

**Ref Item**	Mean	0.060	1.005	2.110	10.904	2.334	0.608*∗*-	3.755	1.498
**1000**	SD	0.010	0.082	0.132	1.064	0.186	0.137	0.257	0.103

**Females**									

**Vehicle**	Mean	0.071	0.751	2.044	6.662	1.805	0.445	0.171	0.696
**0**	SD	0.008	0.066	0.068	0.705	1.186	0.049	0.032	0.177

** 250**	Mean	0.072	0.668	2.010	6.536	1.506	0.500	0.163	0.593
	SD	0.009	0.207	0.121	0.591	0.103	0.098	0.015	0.101

** 500**	Mean	0.070	0.729	2.036	6.263*∗*-	1.498	0.457	0.173	0.625
	SD	0.012	0.079	0.119	0.664	0.152	0.065	0.022	0.109

** 1000**	Mean	0.077*∗*+	0.779	2.091	7.074	1.607	0.474	0.179	0.637
	SD	0.011	0.054	0.116	0.681	0.154	0.056	0.031	0.159

**Ref Item**	Mean	0.078	0.764	2.066	7.023	1.541	0.481	0.178	0.710
**1000**	SD	0.016	0.076	0.132	0.950	0.173	0.077	0.038	0.223

N= number of animals;  ^**∗**+/**∗**-^= statistically significant increase/decrease as compared to vehicle control (p<0.05);  ^**∗∗**+/**∗∗**-^**=** statistically significant increase/decrease as compared to vehicle control (p<0.01); Ref, reference.

**Table 14:** Summary of relative organ weights (organ:body) for the 90-day study on curcumin.

**Group / Dose ** **(mg/kg b.w./day)**		**Fasted B. wt. (g)**	**Relative Organ Weights (%)**									
**n=20**			**Adrenal glands**	**Heart**	**Brain**	**Lungs**	**Liver**	**Kidneys**	**Spleen**	**Thymus**	**Testes/Ovaries**	**Epididymides/Uterus**

	**Males**											

**Vehicle**	Mean	380.079	0.015	0.272	0.557	0.506	2.783	0.604	0.176	0.100	0.969	0.383
**0**	SD	36.908	0.002	0.025	0.048	0.096	0.201	0.039	0.027	0.029	0.107	0.031

**250**	Mean	365.490	0.016	0.283	0.599	0.529	2.843	0.640*∗*+	0.167	0.104	1.039	0.412
	SD	34.259	0.002	0.028	0.048	0.114	0.205	0.055	0.020	0.024	0.089	0.038

**500**	Mean	394.160	0.016	0.280	0.566	0.571	2.913	0.661*∗*+	0.184	0.097	0.993	0.393
	SD	28.692	0.002	0.023	0.048	0.161	0.222	0.135	0.033	0.020	0.139	0.033

**1000**	Mean	380.596	0.015	0.276	0.560	0.471	2.946*∗*+	0.631	0.175	0.103	0.982	0.399
	SD	25.771	0.003	0.022	0.038	0.106	0.267	0.043	0.024	0.022	0.092	0.029

**Reference **	Mean	364.692	0.016	0.277	0.583	0.549	3.003*∗∗*+	0.643*∗*+	0.167	0.101	1.035	0.413
**Item** **1000**	SD	34.131	0.003	0.022	0.063	0.160	0.305	0.051	0.036	0.022	0.091	0.039

	**Females**											

**0**	Mean	222.861	0.032	0.337	0.922	0.656	2.988	0.810	0.200	0.148	0.077	0.315
	SD	16.701	0.003	0.021	0.072	0.147	0.215	0.527	0.025	0.022	0.013	0.089

**250**	Mean	216.313	0.033	0.311	0.932	0.777*∗*+	3.029	0.697	0.231*∗∗*+	0.158	0.076	0.275
	SD	14.168	0.004	0.098	0.069	0.218	0.286	0.041	0.039	0.022	0.009	0.050

**500**	Mean	213.676	0.033	0.341	0.956	0.699	2.931	0.701	0.214	0.158	0.081	0.294
	SD	15.403	0.006	0.028	0.067	0.147	0.226	0.046	0.027	0.032	0.012	0.055

**1000**	Mean	228.603	0.034	0.342	0.920	0.762	3.100	0.705	0.208	0.151	0.079	0.281
	SD	21.479	0.004	0.024	0.071	0.221	0.201	0.047	0.021	0.028	0.013	0.077

**Reference **	Mean	221.654	0.035	0.345	0.936	0.687	3.167	0.695	0.217	0.161	0.080	0.323
**Item** **1000**	SD	19.352	0.006	0.023	0.075	0.129	0.295	0.043	0.031	0.028	0.016	0.106

n= number of animals,  ^**∗**+/**∗**-^**=** statistically significant increase/decrease as compared to control-G1 (p<0.05), and  ^**∗∗**+/**∗∗**-^**=** statistically significant increase/decrease as compared to control-G1 (p<0.01).

**Table 15:** Summary of gross pathology findings in the 90-day study on curcumin.

**Gross Pathology observation (s)**	**Number of animals with or without lesion (s)/ Numbers of animals observed**				
	**Vehicle** **0**	**250**	**500**	**100**	**Ref Item ** **1000**
**Males**					

No abnormalities detected	19/20	18/20	18/20	19/20	13/20

Lungs- Discoloration, yellow	0/20	1/20	1/20	1/20	7/20

Testes- Small sized, unilateral/ bilateral	1/20	0/20	1/20	0/20	0/20

Epididymides- Small sized, unilateral	1/20	0/20	0/20	0/20	0/20

Epididymides- Foci, white	0/20	0/20	1/20	0/20	0/20

Kidneys- Hydronephrosis, unilateral	0/20	1/20	0/20	0/20	0/20

Kidneys- Cystic, unilateral	0/20	0/20	1/20	0/20	0/20

**Females**					

No abnormalities detected	19/20	19/20	20/20	18/20	19/20

Lungs- Discoloration, yellow	0/20	1/20	0/20	2/20	1/20

Kidneys- Cystic, unilateral	1/20	0/20	0/20	0/20	0/20

**Table 16:** Summary of relevant^#^ histopathological findings.

**Organs/Histopathology**	**lesions / no. of tissues examined**				
	**Vehicle** **0**	**250** **∗**	**500** **∗**	**1000**	**ref item 1000**
**Males**					

**Kidneys**					
Basophilic tubules	0/20	- -	- -	1/20	1/20

**Lungs**					
Infiltration, foamy macrophages, alveolar	8/20	7/20	11/20	4/20	11/20
Inflammatory foci, chronic	4/20	1/20	1/20	0/20	0/20
Inflammation chronic and fibrosis, interstitial	0/20	1/20	7/20	3/20	11/20
Granuloma, foreign body	0/20	1/20	5/20	1/20	11/20
Osseous metaplasia	2/20	0/20	0/20	0/20	1/20

**Stomach**					
Erosion, mucosa, glandular, focal	1/20	- -	- -	0/20	1/20

**Spleen**					
Increased extramedullary hematopoiesis	0/20	- -	- -	1/20	1/20

**Thymus**					
Hyperplasia, epithelial	2/20	- -	- -	0/20	1/20

**Testes**					
Atrophy/ degeneration, seminiferous tubules, unilateral/ bilateral	2/20	- -	- -	1/20	0/20

**Epididymides**					
Vacuolation, epithelial	2/20	- -	- -	1/20	1/20
Sperm granuloma	2/20	- -	- -	2/20	1/20
Oligospermia	2/20	- -	- -	1/20	0/20

**Prostate**					
Infiltration, mononuclear cells, interstitial	1/20	- -	- -	1/20	2/20

**Females**					

**Liver**					
Vacuolation, cytoplasmic, periportal	0/20	- -	- -	1/20	2/20

**Lungs**					
Infiltration, foamy macrophages, alveolar	12/20	11/20	10/20	11/20	6/20
Inflammatory foci, chronic	1/20	4/20	1/20	0/20	2/20
Inflammation chronic and fibrosis, interstitial	0/20	4/20	1/20	8/20	4/20
Granuloma, foreign body	0/20	3/20	1/20	2/20	3/20
Infiltration, polymorphonuclear cells, alveolar	0/20	2/20	0/20	2/20	0/20
Osseous metaplasia	1/20	0/20	1/20	0/20	0/20

**Ovaries**					
Cyst, luteal	1/20	- -	- -	1/20	0/20

**Uterus with cervix and vagina**					
Increased mucification, epithelium, cervix and vagina	0/20	- -	- -	1/20	1/20

**Urinary bladder**					
Infiltration, mononuclear cells, submucosa	0/20	- -	- -	1/20	1/20

^#^Findings that occurred only in the vehicle control group or only in one animal are not included. Changes observed in other organs were within normal histological range.

## Data Availability

All experimental records, specimens, and data are archived at Vimta Labs Limited, Pre-Clinical Division, Vimta Life Sciences Facility, Plot No. 5, MN Science and Technology Park, Genome Valley, Hyderabad 500 078, India.
